# A new look at imines and their mixture with PC_71_BM for organic, flexible photovoltaics

**DOI:** 10.1038/s41598-023-38978-x

**Published:** 2023-08-14

**Authors:** Krzysztof A. Bogdanowicz, Sebastian Lalik, Paulina Ratajczyk, Andrzej Katrusiak, Piotr Krysiak, Agnieszka I. Pawłowska, Monika Marzec, Agnieszka Iwan

**Affiliations:** 1 Military Institute of Engineer Technology, Obornicka 136, 50-961 Wrocław, Poland; 2 Institute of Physics, Jagiellonian University, Łojasiewicza 11, 30-348 Kraków, Poland; 3 Faculty of Chemistry, Adam Mickiewicz University, Uniwersytetu Poznańskiego 8, 61-614 Poznań, Poland

**Keywords:** Chemistry, Optics and photonics

## Abstract

Due to its high electron affinity and electron mobility in a wide absorption range of the visible solar spectrum, [6,6]-phenyl-C71-butyric acid methyl ester (PC_71_BM) is often used as an efficient acceptor in organic photovoltaics. In turn, imines are additives to the active layer of organic solar cells, mainly due to the free electron pair of the imine nitrogen atom and the presence of various chemical groups affecting the polarity and conformations of molecules. However, the attainable efficiency is not as high as expected. Therefore, we have systematically investigated two imines and their mixtures with PC_71_BM by spectroscopic (the high pressure UV–Vis and frequency domain dielectric), thermoelectric, and mechanical methods for organic, flexible photovoltaics. Both the imines, (N,NʹE,N,NʹE)-N,Nʹ-([2,2ʹ:5ʹ,2ʺ-terthiophene]-5,5ʺ-diylbis(methanylylidene))bis(benzo[d]thiazol-2-imine) (SC3) and (6E)-N-((5-(5-(5-((E)-(4-(4-(4-fluorophenyl)thiazol-2-yl)phenylimino)methyl)thiophen-2-yl)thiophen-2-yl)thiophen-2-yl)methylene)-4-(4-(4-fluorophenyl)thiazol-2-yl)benzenamine (SC13), have the same core composed of three thiophene rings but different terminal chains of the molecules. In the imine SC3, the imine bond is followed by benzothiazole rings on both sides of the core, while in SC13, a thiazole ring separates two benzene rings, the terminal one F-substituted. The difference in molecular structure affects the electric properties of the neat imine and its mixed layers. An addition of PC_71_BM to the imines improves their electric conductivity. The mechanical studies focused on the stress at break and elongation showed superior behaviour compared to fullerene derivative. High pressure systematically reduces the band gap energy, *E*_*g*_, from 1.68 eV at 0.16 GPa to 1.51 eV at 2.69 GPa for PC_71_BM, from 1.77 eV at 0.1 MPa to 1.53 eV at 4.15 GPa for SC3, and from 1.99 eV at 0.11 GPa to 1.8 eV at 3.10 GPa for SC13, as determined by the UV–Vis absorbance measurements in a diamond-anvil cell. These *E*_*g*_ reductions reflect the compressed intermolecular interactions that can be used to monitor the structural stability of these compounds. Based on the dielectric studies it was found that the relaxation processes registered for both imines are probably the grain boundary relaxation. Two processes also appear in the systems with PC_71_BM, but none of them is the one characteristic of imines. The high-frequency process has a dipole character while the low-frequency one is probably the grain boundary relaxation of these systems. The mechanism of quasi-DC conduction in various temperature ranges in the studied systems was also determined.

## Introduction

Organic compounds are widely investigated for over 30 years for application in solar cells, light emitting diodes, memory devices, transistors or sensors^1^. However, at the present time, the view on organic materials should be extended to a multi-faceted one, taking into account both material, technical and ecological issues. Considering the latest trends in the development of flexible organic photovoltaics, it can be said that efficiency, stability and cost are still issue for practical use. Synergy between materials–device architecture–properties is the key to achieving this goal.

As an flexible substrate for organic devices mainly polyethylene terephthalate (PET) has attracted considerable attention due to its good mechanical properties and applicability^2–4^. In our previous study we described the influence of PTB7 (poly(4,8-bis[(2-ethylhexyl)oxy]benzo[1,2-b:4,5-bʹ]dithiophene-2,6-diyl-alt-3-fluoro-2-[(2-ethylhexyl)carbonyl]thieno[3,4-b]thiophene-4,6-diyl)) on ITO-coated PET substrate. The mechanical properties showed 0.8% elongation between single and double PTB7 layers. The plastic deformation observed on the flexible PET/ITO substrate was 93.1 ± 7.9 MPa, with a break point of 155.4 ± 7.9 MPa, which is an improvement compared to the neat PET/ITO substrate^5^.

To date, no detailed mechanical studies of imines on a flexible substrate have been performed. There are reports in the literature that imine is an organic functional group that can be used to form polymer structures or as a secondary modification of already existing polymers. Snyder et al. presented the formation of imine bonding in order to crosslink the polyester as pre polymer. The imine linkage showed a certain flexibility allowing to recover its tensile properties after the exposure to external force^6^. Very similar observations were noticed for the bio-polymer of fatty acid dimer, proving that the imine linkage could be a good candidate for elastic organic devices^7^. In the other work, elastomers using imine crosslinkers reached the values of breaking strain of 53−451%. Additionally, the samples were able to regain their original shape after tensile force (reaching even large deformation) and exhibited little hysteresis loss during the loading − unloading cycle^8^. In the case of grafted polysiloxanes apart of the aromatic imine as pendent groups authors used coordination crosslinking effect with transition metals. It was found that the polymer with 10 mol% of imine group with iron chloride additive (P1–10%-Fe-0.33) gave the best overall performance, reaching 4.69 MPa of tensile strength and 742% of elongation at break^9^.

On the other hand, among the wide group of new organic compounds considered as donors or acceptors in solar cells also imines called azomethines are investigated by some scientific groups^10–20^. However, their power conversion efficiency (PCE) value for organic solar cells with imines is not higher than 5.5%. It should be stressed that imines were successfully used in perovskite solar cells by the Bogdanowicz et al.^21^ who obtained PCE = 14.4% and Petrus et al.^22^ who attained PCE = 11% for symmetric imines. In our recent paper, we showed the influence of imine SC2 on the dielectric properties of the donor–acceptor P3HT (poly(3,4-ethylenedioxythiophene):PC_61_BM ([6,6]-phenyl-C61-butyric acid methyl ester) system by replacing PC_61_BM with imine SC2 or adding imine SC2 to this system. The relaxation processes connected with the charge movements and with the dipolar rotation (β relaxation) in the systems based on P3HT were revealed. Moreover, it was shown that the addition of imine SC2 to P3HT decreases the specific electric conductivity by an order of magnitude while the conductivity is even lower in the P3HT:SC2:PC_61_BM system^23^.

It should be stressed here, that the nitrogen atom in the imine bond shows hybridization of sp^2^, while the difference in electronegativity of carbon (2.55) and nitrogen (3.04) atoms according to Pauling is about 0.5. Therefore, the properties of imines are not determined mainly by the polarity of the imine bond, but by the free electron pair of nitrogen atom, and the presence of various chemical groups^24^. In addition, it should be emphasized that the imine bond usually occurs in the form of trans configuration and is often obscured by phenyl rings. Furthermore, the presence of various substituents of a donor or acceptor influences the twisting of phenyl rings relative to the imine bond.

Thermoelectric properties of organic devices are important features because they provide information how the temperature or potential value affect the operational features of organic layers. A comprehensive discussion of thermoelectric properties for a series of imines (eight symmetrical and unsymmetrical molecules) was presented^25^. In the same work the influence of certain additives such as camphorosulfonic acid (CSA), PTB7 and/or PC_71_BM ([6,6]-phenyl-C71-butyric acid methyl ester) and P3HT were described. It was observed that the binary mixture SC13:PC_71_BM (1:1, w/w) prepared on a glass support (spin-coating, 5000 rpm) improved thermoelectric properties by a decrease of resistance from 113.8 to 96.2 Ω, without any signs of decomposition. The resistance increased during self-heating as a consequence of the increase in potential. So far, no studies have been carried out on thin layers of samples prepared at a lower rotational speed on a PET/ITO substrate.

Additionally, high-pressure studies provide the information about the potential improvement or deterioration of the photovoltaic properties in connection to the energy gap changes in the modified structures^26–29^. In most cases, the elevated pressure reduces the intermolecular and interionic distances and increases the mobility of electrons, although the reverse processes are also possible^26–30^. Furthermore, the compression provides the information about the performance of the thin photovoltaic layers under strain^5^.

Presently various acceptor materials are used as component of the active layer of organic solar cells, however, PC_71_BM is investigated far less intensively than PC_61_BM. A key shape difference between these two novel organic semiconductors envisaged for photovoltaic applications is the longer ellipsoidal molecule of PC_71_BM compared to the more spherical PC_61_BM molecule^31^. In PC_71_BM material energetic transitions are possible, while they are forbidden in PC_61_BM. Moreover, the presence of PC_71_BM improves the absorption characteristics compared to PC_61_BM for the visible range of the solar spectrum and has a positive effect for photon harvesting. Finally, solar cells with PC_71_BM should exhibit a potentially higher photocurrent than the devices with PC_61_BM. In our last work, we investigated the effect of various small unconjugated aromatic rings attached to the surface of C_70_ fullerene on the selected chemical and physical properties, affecting their suitability for use in solar cells as the electron acceptors in active layers^32^. It was found that the investigated fullerene derivatives exhibit much better electrical properties than PC_71_BM, however still photovoltaic parameters, such as the power conversion efficiency (PCE) in the solar cells with active layer based on PTB7 and other new fullerene derivatives, was low. The next stage of research should be to determine the relationship for non-fullerene organic solar cells on a flexible substrate for practical applications. It should be stressed that although the non-fullerene acceptors exhibit favorable photoelectric properties and high efficiency in the organic solar cells, they are relatively sensitive in the thick film due to the lower electron mobility and anisotropic charge transport. The most popular is commercially available Y6 called also BTP-4F ((2,20-((2Z,20Z)-((12,13-bis(2-ethylhexyl)-3,9-diundecyl-12,13-dihydro-[1,2,5]thiadiazolo[3,4-e]thieno[2ʺ,30ʹ:4ʹ,50]thieno[20,30:4,5]pyrrolo[3,2-g]thieno[20,30:4,5]thieno[3,2-b]indole-2,10-diyl)bis(methanylylidene))bis(5,6-difluoro-3-oxo-2,3-dihydro-1H-indene-2,1diylidene))dimalononitrile)). Organic solar cells based on Y6 as the electron acceptor and polymer PBDB-T-2F (PM6, poly[(2,6-(4,8-bis(5-(2-ethylhexyl-3-fluoro)thiophen-2-yl)-benzo[1,2-b:4,5-bʹ]dithiophene))-alt-(5,5-(1’,3’-di-2-thienyl-5’,7’-bis(2-ethylhexyl)benzo[1ʹ,2ʹ-c:4ʹ,5ʹ-cʹ]dithiophene-4,8-dione)]) as the electron donor exhibited power conversion efficiency PCE = 15.7%^33^. Among other non-fullerene acceptors there are ITIC, N3 (Y6-N3), TPT10 and Y7. In organic solar cells also a binary acceptor system with N3:PC_71_BM and PBDB-T-2F (PM6) as the polymer donor was used and devices exhibited PCE = 16.74% and 18.69% while D18-Cl:N3:PC_61_BM was used as the active layer materials^34^. Although currently fullerene-free organic solar cells have great application potential as large-area, flexible PV, there are still some issues that should be clarified and regulated (e.g. cost, stability, ecological aspects, green solvents, no use of inorganic catalysts, electric properties, and morphology on large, flexible substrate)^34,35^.

Considering the above, we are interested in investigating the potential impact of the imines and imine:PC_71_BM mixture on their properties by means of high pressure UV–Vis spectroscopy, applied for the first time, towards the improvement of the electrical properties of these compounds. Moreover, we extensively investigated the properties of imines and imine:PC_71_BM compositions by Frequency Domain Dielectric Spectroscopy (FDDS). Finally, toward practical aspects of our study, simple flexible organic devices on the PET substrate were constructed and investigated by thermographic camera and mechanical method.

## Experimental

### Materials

Synthesis of SC3 and SC13 was recorded as described in our previous papers^10,36^. [6,6]-Phenyl-C71-butyric acid methyl ester (PC_71_BM) was purchased from Ossila. Chlorobenzene (pure) was purchased from Avantor Performance Materials Poland S.A. PET/ITO with 10–14 sheet resistance, opaque < 3% and transparency 70–72% was purchased from 3D-nano (Kraków, Poland).

### Methods

High-pressure UV-Vis spectra were collected for PC_71_BM, SC3, SC13 and SC3:PC_71_BM and SC13:PC_71_BM mixtures in 1,2-dichlorobenzene (DCB). A modified Merrill-Basset anvil cell (DAC) equipped with low-fluorescence synthetic AII diamond culets was used for generating pressure^37^. The samples were deposited by pressing the small amount of the sample into the diamond culet using a glass plate to form a uniform layer or by applying a drop of solution of the study compound and evaporating the solvent. Glycerol was used as the pressure-transmitting medium. We also measured absorption spectra for the solutions under high-pressure. In all experiments, the gaskets were made of tungsten foil, 0.1–0.2 mm thick with sparked-eroded hole of 0.4 mm in diameter. The pressure was determined by the emission of ruby chip^38,39^. High-pressure UV-Vis spectra were measured using Jasco V-650 and Jasco V-770 at scan speed of 200 nm min^−1^.

Thermal behaviour was acquired using an IR camera (VIGOcam v50, VIGO System S.A, Poland), while applying bias voltage between 0 and 10 V and using a multichannel potentiostat galvanostat (PGStat Autolab M101, Metrohm, Nederland) connected to computer. This experimental procedure was described in details in our previous work^40^. The voltage applied in the experiment was in the range from 0 to 10 V in steps of 0.5 V; each voltage value was maintained for 3 min. The current response was recorded during all these 3-min periods and in the last 10 s the IR image was collected. The work of both camera and power source was controlled via computer software. For the experiment the active layers from solution in dichloroethane with total concentration 15 mg/mL were prepared by spin-coating method at 900 rpm for 20 s. The samples for thermal studies were prepared in a form of a sandwich like-structure between two ITO-coated PET supports giving the total area of approximately 0.18 cm^2^.

The mechanical properties (tensile strain) were investigated on an Instron 33R4469 (Instron, Norwood, MA, USA) testing machine with a load cell of 5 kN, and the results were registered using Bluehill 3.0 software (Instron, Norwood, MA, USA). Compound solution (10 mg/mL in chlorobenzene) was spun cast (900 rpm, 30 s) on a 5 × 5 cm^2^ ITO-coated PET support. For mechanical tests, 5 × 1 cm^2^ plates were used.

Dielectric studies (frequency domain dielectric spectroscopy, FDDS) were performed by using a broadband dielectric Novocontrol Alpha spectrometer (Novocontrol Technologies GmbH & Co. KG, Montabaur, Germany). All samples in the form of thin pellets (tightly compacted crystallites) were placed between two brass electrodes. The thickness of the pellets was measured with a micrometer screw (see Table 1). The geometry of the dielectric measurement setup was the same as in our recent study^23^. After the measurements of SC13 and SC3 samples, the imines were recovered for use as additives to PC_71_BM. The two-component systems PC_71_BM:SC13 and PC_71_BM:SC3 were prepared in a weight ratio of 8:1 (as it was in our previous paper^10^) with a final concentration of PC_71_BM:imine of 25 mg/mL. In the case of pristine imines SC13 and SC3, samples were prepared in an ambient atmosphere, while samples based on PC_71_BM in a glove box in an argon atmosphere.

**Table 1:** Thickness of the samples measured by frequency domain dielectric spectroscopy.

Sample	d [mm]
SC13	0.17
SC3	0.41
PC_71_BM	0.25
SC13:PC_71_BM	0.20
SC3:PC_71_BM	0.28

All samples were subjected to heating—cooling—heating cycle prior to the actual study: (1) heating from room temperature up to 220 °C, (2) cooling to 25 °C and (3) heating up to 220 °C, all at 10 °C/min. The actual measurement was performed during cooling in the temperature range of 220–0 °C in steps of 4 °C, with a measuring voltage of 0.1 V, in the frequency range of 0.01 Hz to 10 MHz for pristine imines SC3 and SC13. Due to fluctuations in the relationships εʹ(ν) and εʺ(ν) in the low frequency range for the PC_71_BM sample in the high temperature range (220–200 °C), the measuring frequency range was limited to 1 Hz to 10 MHz for all the samples based on PC_71_BM. Before each measurement, the temperature was stabilized with an accuracy better than ± 0.5 °C.

## Results and discussion

### Base characteristic of imines

**Figure 1:**
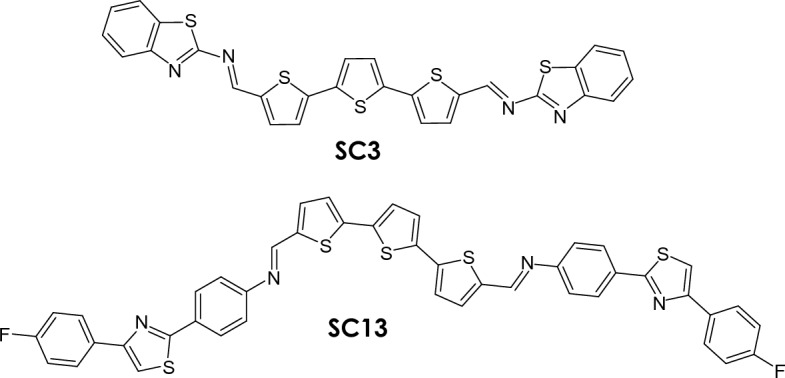
Chemical structure of investigated imines.

Both imines (N,NʹE,N,NʹE)-N,Nʹ-([2,2ʹ:5ʹ,2ʺ-terthiophene]-5,5ʺ-diylbis(methanylylidene))bis(benzo[d]thiazol-2-imine) (SC3) and (6E)-N-((5-(5-(5-((E)-(4-(4-(4-fluorophenyl)thiazol-2-yl)phenylimino)methyl)thiophen-2-yl)thiophen-2-yl)thiophen-2-yl)methylene)-4-(4-(4-fluorophenyl)thiazol-2-yl)benzenamine (SC13) were fully characterized previously^10,36^. Briefly, the ^1^H NMR spectra of both imines showed characteristic peak due to HC = N– proton at 8.62 ppm for SC3 and 8.56 ppm for SC13. In the FTIR spectra the bands at 1659 cm^−1^ (SC3) and 1610 cm^−1^ (SC13) corresponding to HC=N–stretching were found. Imines SC3 and SC13 exhibited T_5%_ weight loss at 303 and 390 °C with residue at 800 °C—67% and 49% as was checked by thermogravimetric analyses (TGA) under nitrogen atmosphere with heating rate of 5 °C/min. HOMO and LUMO levels investigated via cyclic voltamperometry in dicholomethane solution were found at − 5.07 eV and − 2.57 eV for SC3 and at  − 6.11 eV and  − 3.80 eV for SC13, corresponding to energy gap *E*_g_ equal to 2.50 eV and 2.31 eV, respectivly. Chemical structures of both imines are presented in Fig. 1.

### High pressure spectroscopy of imines and their mixtures with PC_71_BM

**Figure 2:**
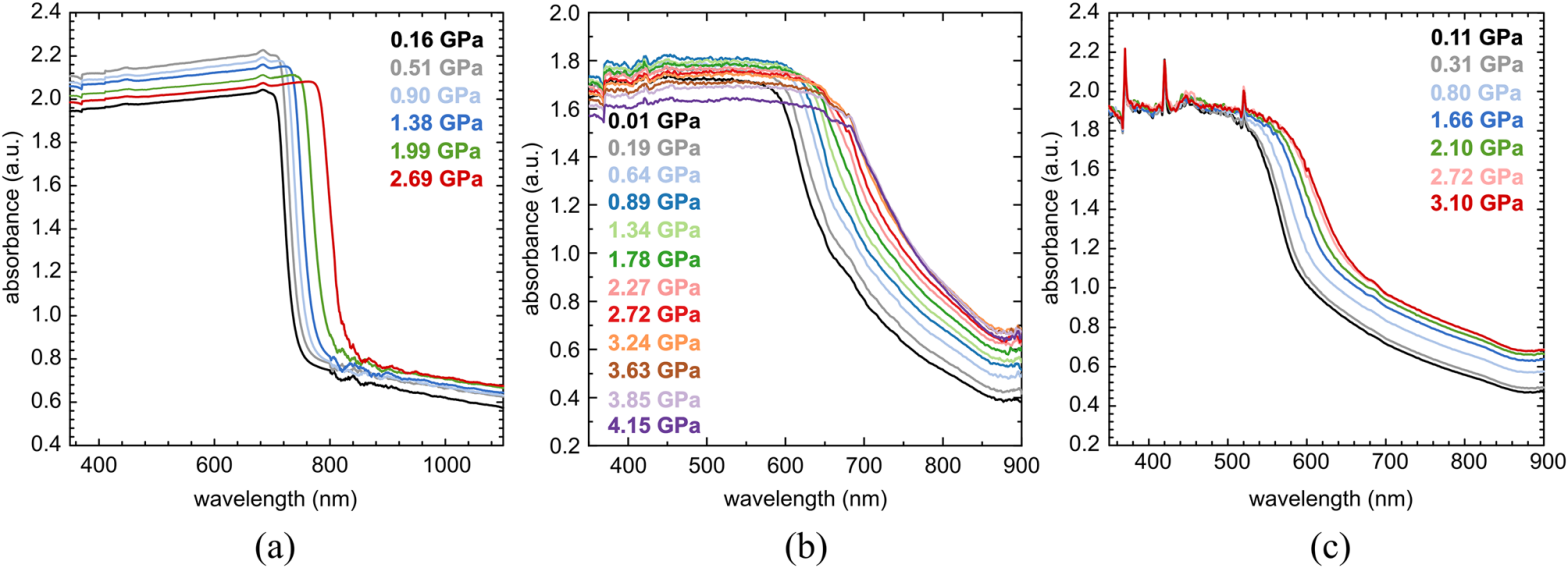
High-pressure absorbance spectra of (**a**) PC_71_BM; (**b**) SC3; and (**c**) SC13. Measured samples were prepared by pressing the crystalline powder into a diamond culet and forming a thin (2–3 μm) uniform film.

**Figure 3:**
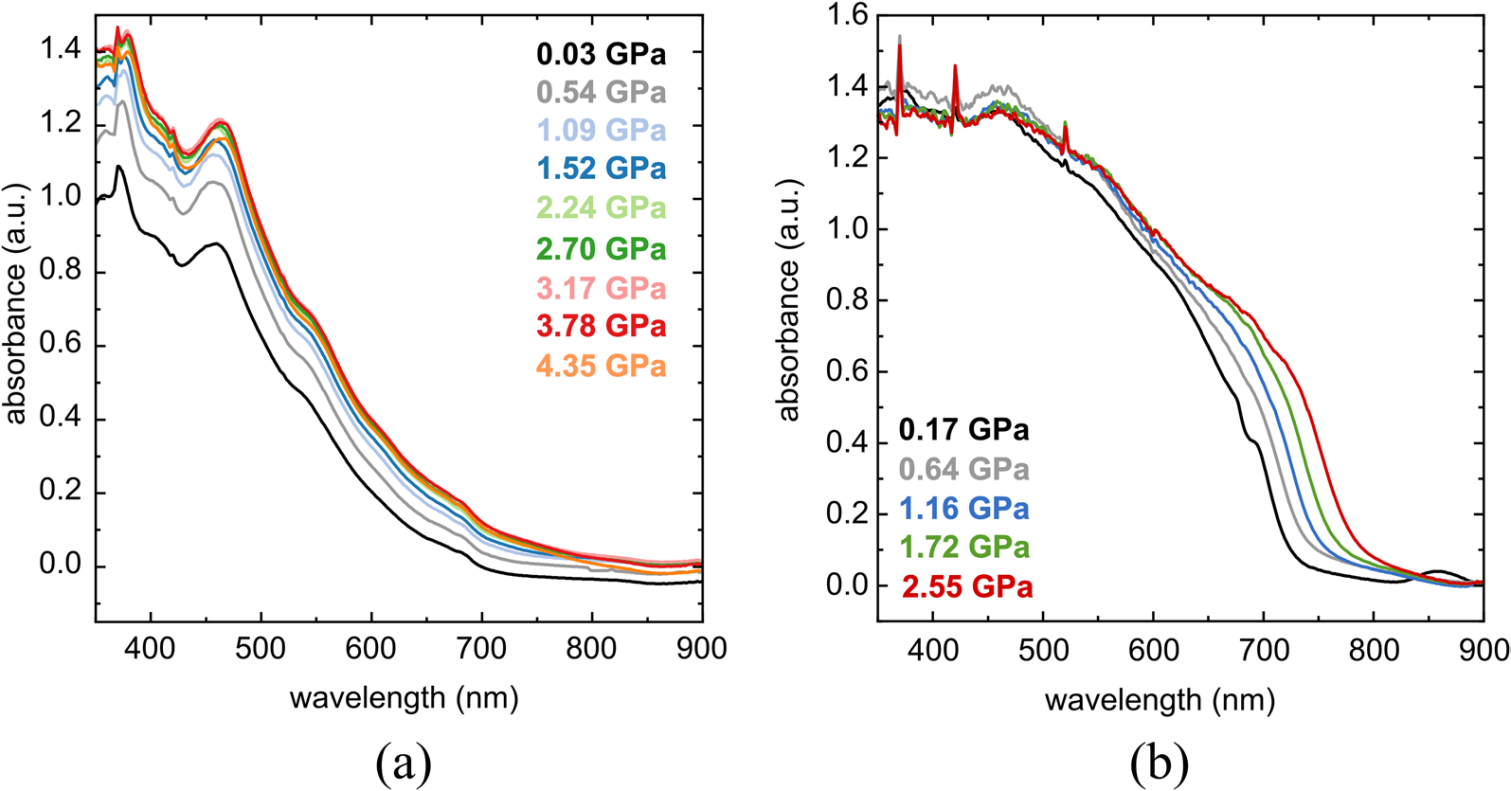
Absorbance spectra of (**a**) SC3:PC_71_BM and (**b**) SC13:PC_71_BM layers upon compression. The films were deposited on the diamond culet by placing a drop of mixtures on it and evaporating DCB.

To study the effect of pressure on the electronic band gap, UV-Vis absorption experiments were performed for PC_71_BM, SC3, SC13 as well as SC3:PC_71_BM and SC13:PC_71_BM mixtures compressed in the DAC chamber. The investigated compounds and mixtures were deposited by pressing the small amount of the sample into the diamond culet using a glass plate to form a uniform layer (Fig. 2) or by applying a drop of solution of the study compound and evaporating the solvent (Fig. 3). All three compounds show a bathochromic shift with increasing pressure (Fig. 2), the same as SC3:PC_71_BM and SC13:PC_71_BM mixtures (Fig. 3). The absorption edge in the absorbance spectrum of PC_71_BM is much sharper compared to those in the spectra of SC3 and SC13.

**Figure 4:**
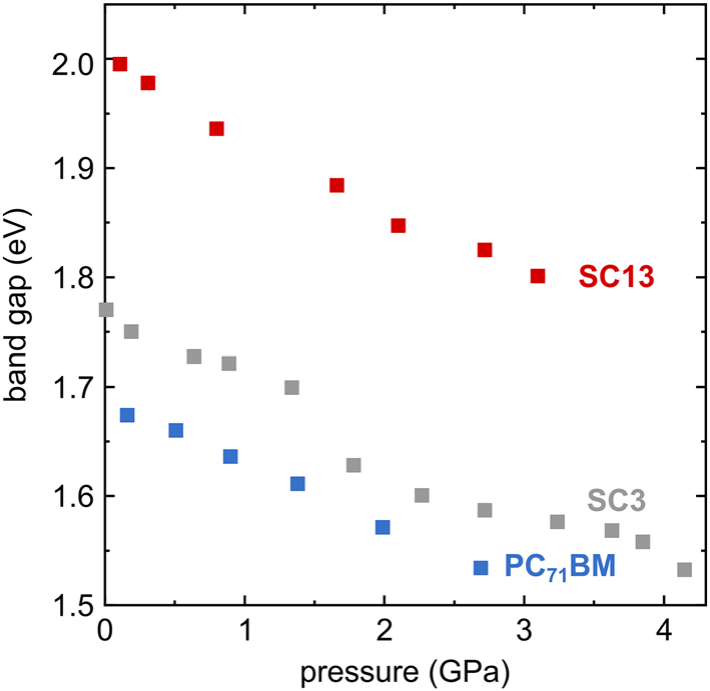
Pressure dependence of the optical energy gap for PC_71_BM, SC3 and SC13.

The correlation between the pressure and the optical band gap (*E*_g_) in three studied compounds PC_71_BM, SC3 and SC13 is shown in Fig. 4. The band gap in PC_71_BM, decreases linearly with increasing pressure from 1.67 eV at 0.16 GPa to 1.53 eV at 2.69 GPa. A similar linear *E*_g_ narrowing from 1.99 eV at 0.11 GPa to 1.8 eV at 3.1 GPa occures in SC13. However, a nonlinear decrease in the band gap is observed in SC3, which may be associated with solid–solid phase transition in this compound. The band gap in SC3 decreases from 1.77 eV at 0.1 MPa to 1.53 eV at 4.15 GPa.

**Figure 5:**
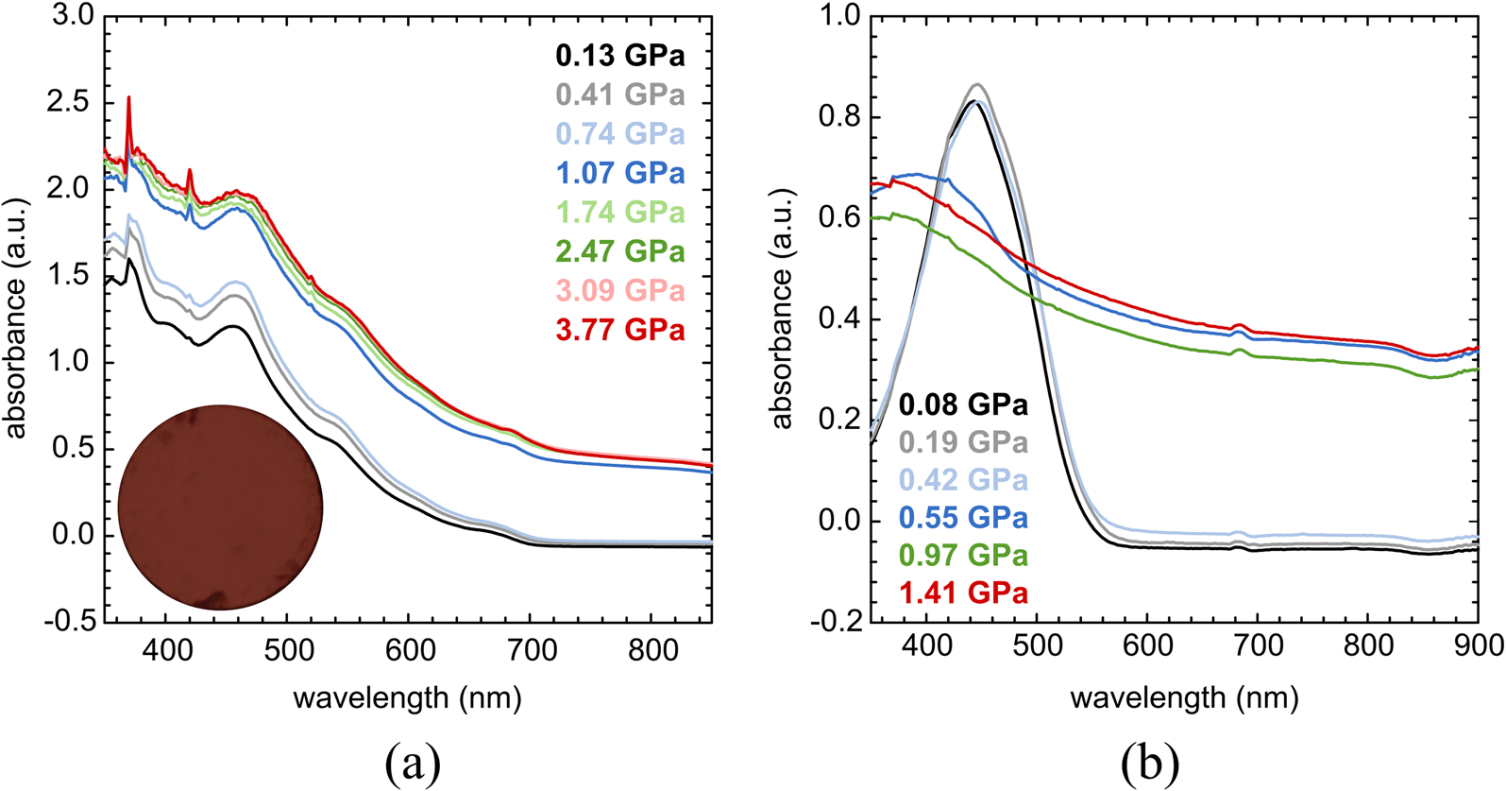
High-pressure absorption spectra for: (**a**) PC_71_BM dissolved in chloroform compressed up to 3.77 GPa; and (**b**) the solution of SC3 in 1,2-dichloroethane compressed up to 1.41 GPa. The PC_71_BM:chloroform solution remained liquid up to 0.74 GPa (*i.e*. approximately the freezing pressure of chloroform at 296 K)^41^; the inset shows the DAC chamber with the solution at 0.13 GPa. The SC3:1,2-dichloroethane solution froze at 0.50 GPa, which is lower than the freezing pressure of the pure 1,2-dichloroethane at 0.7 GPa^42^. We did not observe the precipitation of PC_71_BM or SC3 after the crystallization of the solvents.

As an example, we also measured absorption spectra for solutions at high-pressure for PC_71_BM (Fig. 5a) and SC3 (Fig. 5b) dissolved in chloroform and 1,2-dichloroethane, respectively. For a solution of PC_71_BM in chloroform, there is a noticeable increase in absorbance when the chloroform solution freezes above 0.74 GPa (the pure chloroform freezes at 0.62 GPa at 296 K)^41^. However, for a solution of SC3 in 1,2-dichloroethane, above 0.42 GPa, a decrease in absorbance and a clear change in the shape of the spectrum is noted below the freezing pressure of 1,2-dichloroethane at 0.7 GPa^42^. This decreased absorbance can be due to a drastic decrease in the solubility of SC3 in the pressure range of about 0.4 GPa.

### Thermal IR images of PC_71_BM, imines and their mixture with PC_71_BM on flexible support: normal and pressure effect

The thermal imaging studies are based on the phenomenon of heat generation as a result of electric current conduction through the plane of a layer. In this experiment, organic layers based on three organic compounds, namely: SC3, SC13, and PC_71_BM, in single- and dual-component mixtures were studied. The samples’ architecture was as follows:

- PET/ITO/SC3/Ag(paste)/ITO PET;
- PET/ITO/SC13/Ag(paste)/ITO PET;
- PET/ITO/PC_71_BM/Ag(paste)/ITO PET;
- PET/ITO/SC3:PC_71_BM (1:1)/Ag(paste)/ITO PET;
- PET/ITO/SC13:PC_71_BM(1:1)/Ag(paste)/ITO PET.

The samples were tested under incrementation of external potential from 0 to 10 V in steps of 0.5 V and duration of the interval equals to 3 min. Such experimental methodology ensures the observation of a stable temperature image. The measuring setup allows registration of current passing during whole experiment indicating the stability of conduction process across the sample. The measurements with reverse polarization was not possible to perform because the samples disintegrate after each test.

**Figure 6:**
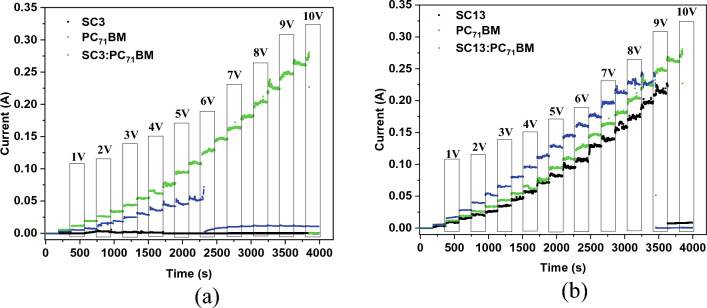
Current evolution over time during the experiment for SC3 (**a**) and SC13 (**b**) sets as well as for PC_71_BM.

In the first step, the correlation of current in time was done to assess the general performance and to observe the current conduction process (Fig. 6).

Figure 6a represents results for the set of SC3 (SC3, PC_71_BM, and SC3:PC_71_BM mixture) which evidently demonstrate that the thin layer containing imine SC3, prepared by the spin-coating technique at 900 rpm exhibits very poor conductivity with maximum current value of 0.0004 A at 2 V. As it can be noted, the conductivity was not completely stable and dropped at higher potentials, above 3 V. The mixture of SC3 and PC_71_BM gave better results than the pristine imine, a stable increment of the current was observed up to 5.5 V. When 6 V was reached, a sudden drop of current was observed and maintained its value around 0.01 A, till 10 V. Only a visible fluctuation was observed at the beginning of new interval. If we compare those results with neat PC_71_BM, it is evident that the conductive properties of fullerene derivative are much better and stable during whole experiment. Those results demonstrate that PC_71_BM influences greatly the conductivity of the mixed layer. The current drop at 6 V might suggest that some processes are taking place within the layer, including a degradation processes.

When analysing the second set of samples based on SC13 and PC_71_BM, prepared in the same way as SC3 set, it turned out that it shows a different behavior. The SC13 imine has a similar conductivity to PC_71_BM, up to 4.5 V. Above this value the current differences are at the level of approx. 0.01 A. After reaching 9 V, the current for SC13 decreases to 0 A, hence the sample is no longer conductive. As for the PC_71_BM, the current value drops at 10 V. In the case of SC13:PC_71_BM (1:1) mixture, the layer shows better performance properties compared to both neat components. Also in the case of the mixed sample, a drop of current was observed at a voltage of 9 V.

**Figure 7:**
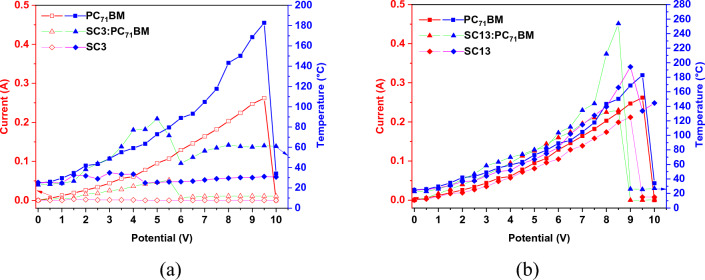
Correlation between current (or temperature) and applied potential for SC3 (**a**) and SC13 (**b**) sets as well as for PC_71_BM.

The correlation between induced current and applied potential presented in Fig. 7, for samples PC_71_BM, SC13, SC13:PC_71_BM, and SC3:PC_71_BM (up to 5.5 V) gave a linear-like tendency, suggesting the conducting type of behaviour. Sheet resistance and conductivity are compiled in Table 2.

**Table 2:** The values of sheet resistance and conductivity for all studied samples.

Sample	Sheet resistance [Ω/cm^2^]	Conductivity [μS/m]
PC_71_BM	365.0	3.38
SC3	3031.5 (at 1.5V)	0.40 (at 1.5V)
SC3:PC_71_BM	1188.4	1.04
SC13	416.8	2.96
SC13:PC_71_BM	186.8	6.61

The sheet resistance values range from 186.8 to 3032 Ω/cm^2^ and temperature range is from 16 to 260 °C. The thermal response of SC13, PC_71_BM, and SC13:PC_71_BM samples is very similar. In the case of samples containing SC3, their behaviour showed low or no temperature generation.

**Figure 8:**
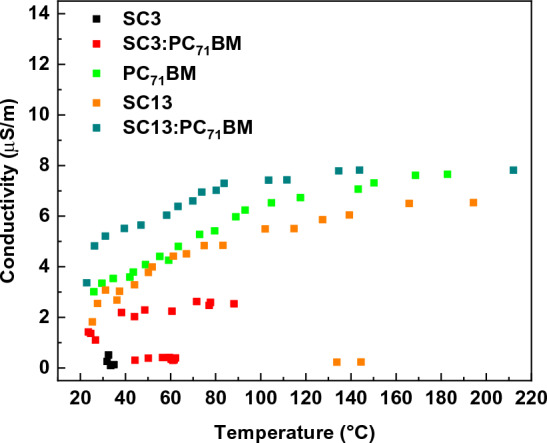
Correlation between conductivity and temperature of all studied samples caused by passing current.

When analysing the electrical and thermal behaviour in relation to increasing potential, it is worth mentioning that the general trend was observed where the increase of temperature coincidences with the increase of current flow. This also applies to the situations where the current drops and the decrease in observed temperature is noticeable. It was observed that for samples whose temperatures exceeded 180 °C, a partial or the complete disintegration of the layers occurred. This can be very easily confirmed when we correlate the temperature of the sample (caused by current flow) with conductivity (Fig. 8). The conductive behaviour of all samples presents a slightly increasing line up to the temperature at which the disintegration of the sample occurs. After the critical temperature was reached, a decrease in conductivity was registered for temperatures below the highest observed, e.g. for SC13 in the temperature range from 130 to 220 °C.

Visual inspection was carried out to confirm the physical disintegration of the samples and for the samples for which the temperature was above approx. 160 °C, deformation of the PET material was observed due to the approach to the melting temperature (approx. 246–256 °C, depending on the manufacturer)^43^. This was not observed for the samples containing SC3 for which the maximum temperature was 90 °C.

**Figure 9:**
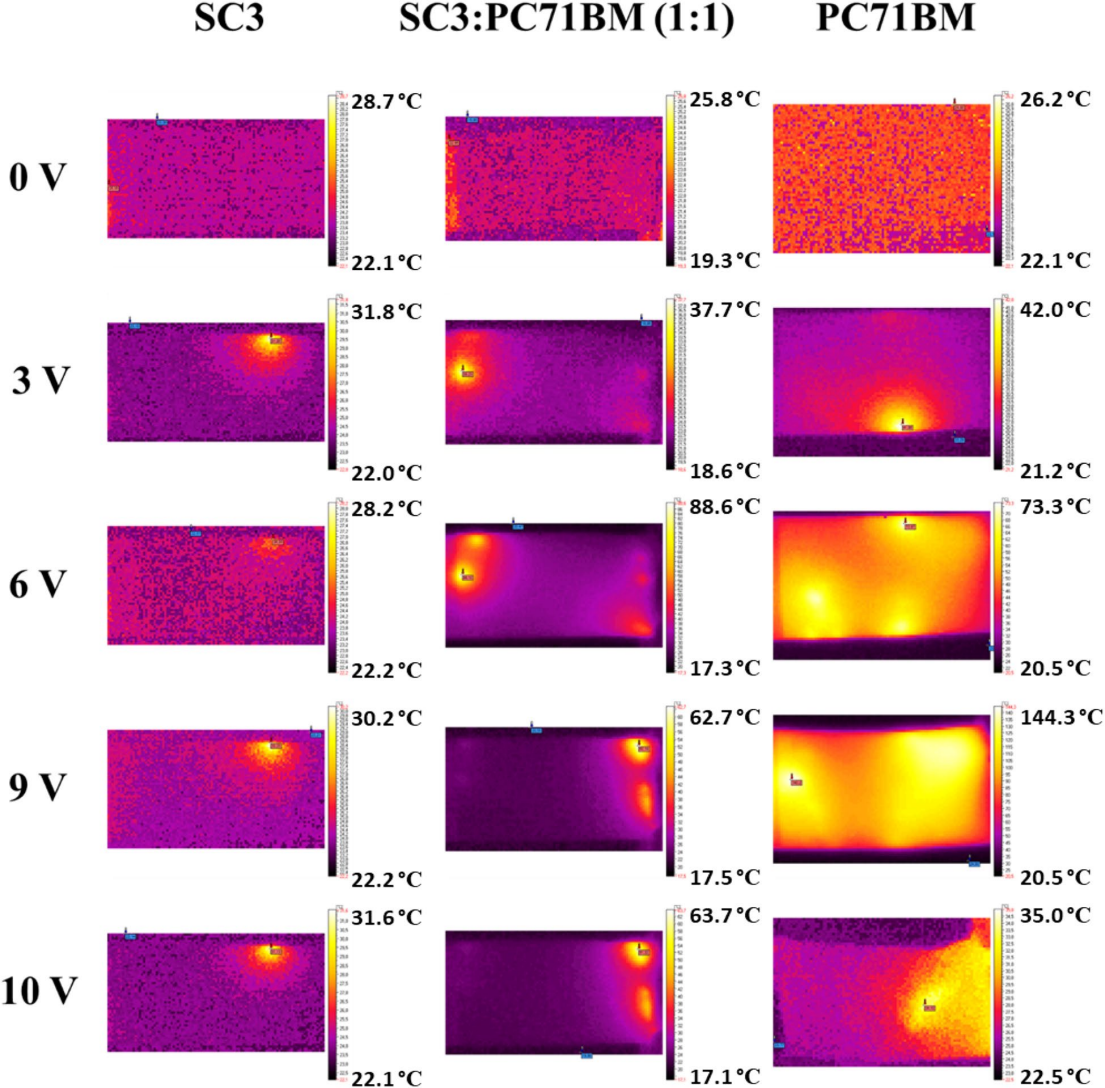
Thermographs registered for 0 V, 3 V, 6 V, 9 V and 10 V for SC3, SC3:PC_71_BM and PC_71_BM. The scale from black to white is individual for each image and represents the highest and the lowest temperature observed.

In order to explain the different behavior of the samples containing the imine SC3, an analysis of thermographs was carried out. Figure 9 presents the images registered for SC3, SC3:PC_71_BM and PC_71_BM samples at selected potentials. It can be seen that the samples containing the imine SC3 show punctate areas where the temperature increased and these spots are most often located at the edges of the sample where the contact is the weakest. After the experiment the sample was completely intact. As expected, SC3:PC_71_BM showed more heating zones which correspond well with the electric data. In the case of PC_71_BM, the heating affected a bigger area than in the case of samples containing imine SC3. The heating zone expands and heating seems to be more uniform. The process of exfoliation of protecting tape used to join both electrodes together was also registered on the thermograms. The image registered for 10 V shows cooling process after the sample was deformed and its continuity was broken.

**Figure 10:**
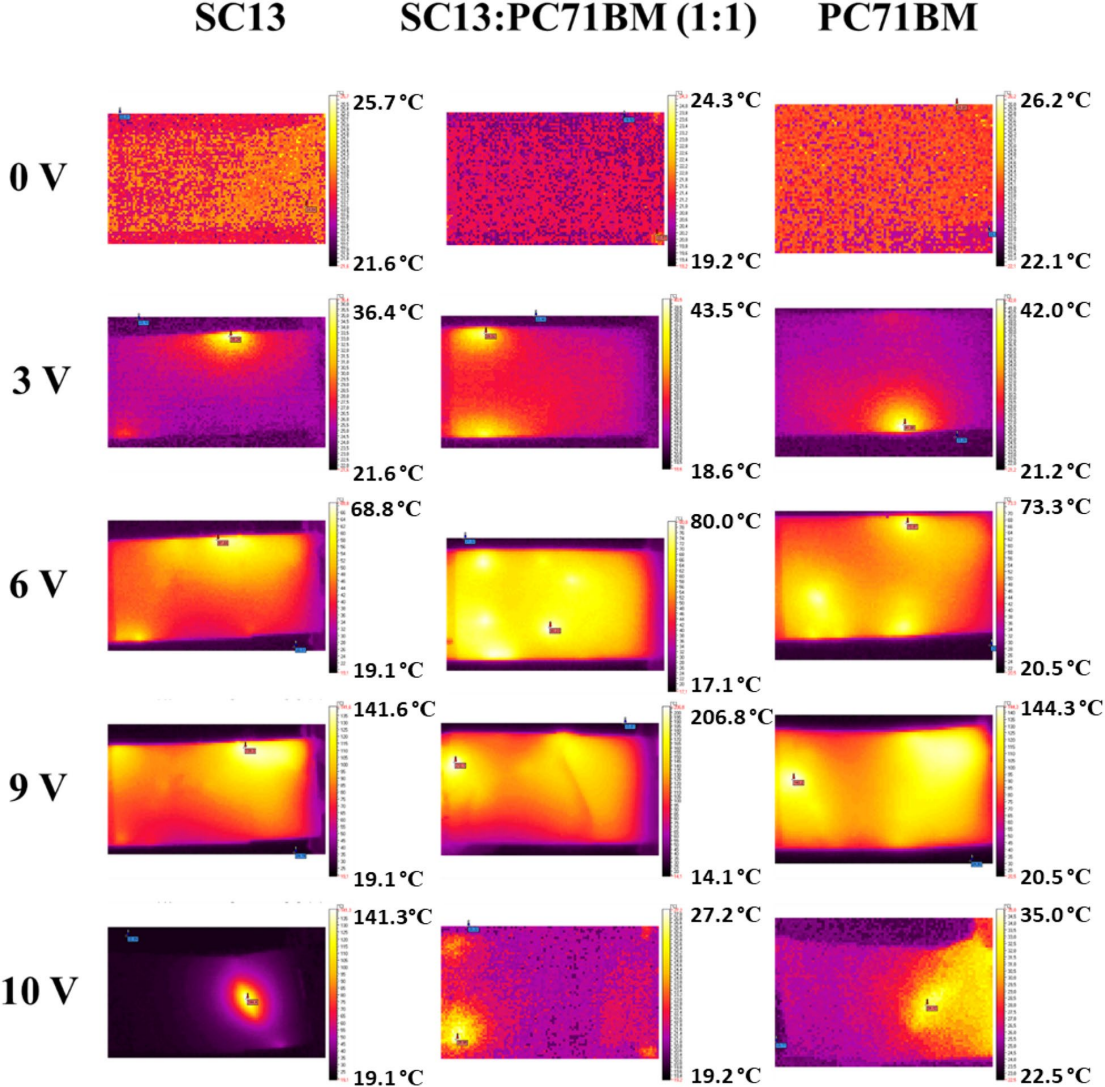
Thermographs registered for 0, 3, 6, 9 and 10 V for SC13, SC13:PC_71_BM and PC_71_BM. The scale from black to white is individual for each image and represents the highest and the lowest temperature observed.

The thermal images registered for imine SC13 are similar to those for PC_71_BM (see Fig. 10). The heating areas cover almost the entire sample, with the SC13:PC_71_BM mixture also showing uniform heating for 6 V of applied potential. This confirms the assumption made on the basis of electrical data. The exfoliation of protecting tape and the distortion of sample geometry are also apparent from compared images for SC13 for 9 and 10 V (Fig. 10).

Additionally, an experiment to evaluate the influence of pressure on the sheet resistance was performed. The sample architecture did not contain silver paste and was focused on compression of PET/ITO covered with organic layer with other PET/ITO support. These measurements showed generally much higher resistances than thermo-electric experiment, probably due to imperfect contact between layers and elastic behaviour of support itself. The resistance values were 2–3 orders of magnitude greater than those presented in Table 2. However, significant error for each sample (1 order of magnitude) suggests that the measurement setup should be adapted to the nature of the support and sample.

In our previous work^10,36^ we have studied the behaviour of SC3 and SC13, with PTB7 or PC_71_BM (only for SC13) on a glass support and the layers were prepared by using the same technique but at 5000 rpm. Those studies also showed a conductive behaviour of all neat samples and their mixtures. The observed ranges of currents were very similar and more uniform heating of the layer was also observed in the case of mixed samples. The difference from this study is the lower thermal resistance of prepared samples and the very low conductivity of imine SC3. Taking into account that imine compounds have relatively high decomposition temperature (approx. 300 °C), the observed disintegration of the sample is most likely related to the kind of used support: ITO on the glass support is more stiff and more thermally resistant than PET. As for the SC3 imine behavior, it might be related to the probably different organization of the molecules in the layer prepared at different revolution per minute (hence slightly increased thickness).

To summarize, imines SC3 and SC13 prepared on the elastic support present electrical properties that can be improved by adding PC_71_BM. The enhancement is most likely related to the molecule rearrangement of both components, resulting in an improvement in the electric conductivity through the layer. The use of thermally sensitive support resulted in samples disintegration at temperatures above 180 °C, which was not observed in the case of samples on a glass substrate.

### Mechanical properties of PC_71_BM, imines and their mixture with PC_71_BM on PET substrate

**Figure 11:**
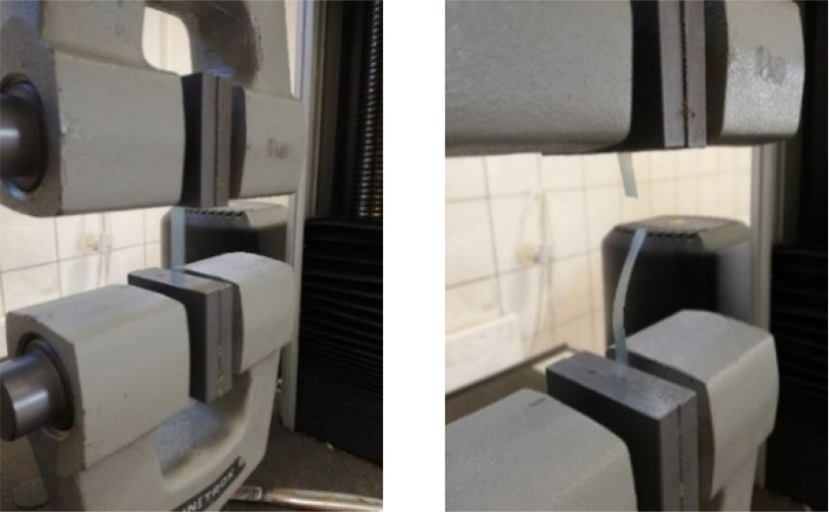
Selected photos of the tested samples during the tear test for PET/ITO/SC3.

In order to evaluate the influence of the organic layer on the substrate, mechanical studies have been performed on samples consisting of PET film covered with ITO and applied with the studied organic layer. As the organic layer, PC_71_BM, SC3, SC13 and binary mixtures with corresponding imine and PC_71_BM were evaluated. The sample thickness was 0.12 or 0.13 mm. Tensile tests were performed in the static mode registering the breaking force and elongation. Figure 11 presents exemplary test results after break. As it was observed for all samples the braking line was uniform and located closer to the moving clamp.

On the basis of the recorded force value and the known cross-section of the samples, the maximum normal stress at break was calculated as:

1 $$\sigma =\frac{{F}_{max}}{A}$$

where: *F*_*max*_—maximum force recorded during stretching, *A*—cross-sectional area of the sample.

The relative elongation for the tested samples was determined based on the following equation:

2 $$\Delta L=\frac{{L}_{k}-{L}_{p}}{{L}_{p}} [\%]$$

where: *L*_*k*_—final length of the sample (at break), *L*_*p*_—initial length of the sample.

The results of mechanical tests for all investigated samples on PET substrate (including multiplications) are summarized in Table 3.

**Table 3:** Results of mechanical testes for all investigated samples on PET substrate.

No of sample	*F*_max_ [N]	*L*_p_ [mm]	*L*_k_ [mm]	Δ*L* [%]	Thickness [mm]	Width [mm]	*A* [mm^2^]	σ [MPa]
**PET/ITO/SC3**								
A	240.7	25.0	45.8	83	0.130	9.61	1.25	192.7
B	247.9	25.0	46.2	85	0.130	9.64	1.25	197.8
C	270.6	25.0	45.0	80	0.130	10.39	1.35	200.3
D	240.1	25.0	43.5	74	0.130	10.18	1.32	181.4
E	253.4	25.0	44.0	76	0.130	10.32	1.34	188.9
Average value (ESD)	**251(13)**			**80(5)**				**192(8)**
**PET/ITO/SC13**								
A	250.6	25.0	44.0	76	0.130	9.68	1.26	199.1
B	239.1	25.0	44.0	76	0.130	9.69	1.26	189.8
C	238.5	25.0	46.0	84	0.130	9.52	1.24	192.7
D	233.3	25.0	42.0	68	0.130	9.97	1.30	180.0
E	255.6	25.0	44.0	76	0.130	10.34	1.34	190.2
Average value (ESD)	**243(9)**			**76(6)**				**190(7)**
**PET/ITO/PC**_**71**_**BM**								
A	231.3	25.0	39.8	59	0.130	9.7	1.26	183.4
B	232.9	25.0	43.0	72	0.130	9.3	1.21	192.6
C	225.7	25.0	39.9	60	0.130	9.49	1.23	182.9
D	246.7	25.0	39.8	59	0.130	10.42	1.35	182.1
E	234.2	25.0	39.6	58	0.130	9.82	1.28	183.5
Average value (ESD)	**230(4)**			**64(7)**				**186(6)**
**PET/ITO/SC3:PC**_**71**_**BM**								
A	220.9	25.0	39.2	57	0.120	9.63	1.16	191.2
B	252.9	25.0	44.1	76	0.120	9.78	1.17	215.5
C	244.0	25.0	44.1	76	0.120	9.58	1.15	212.2
D	248.2	25.0	44.2	77	0.120	9.76	1.17	211.9
E	236.8	25.0	42.0	68	0.120	9.91	1.19	199.1
Average value (ESD)	**241(13)**			**71(9)**				**206(10)**
**PET/ITO/SC13:PC**_**71**_**BM**								
A	229.4	25.0	41.5	66	0.120	9.62	1.15	198.7
B	253.6	25.0	46.4	86	0.120	9.77	1.17	216.3
C	226.6	25.0	40.5	62	0.120	9.78	1.17	193.1
D	221.0	25.0	40.8	63	0.120	9.51	1.14	193.7
E	240.3	25.0	42.0	68	0.120	10.1	1.21	198.3
Average value (ESD)	**234(13)**			**69(10)**				**200(10)**

The estimated standard deviations (ESD) refer to the last significant places of the *F*_max_ values, shown in bold.

The samples showed the value of the stress at break between (186 ± 5.5) and (206.0 ± 10.4) MPa and the relative elongation ranging from (64 ± 7)% to (80 ± 5)%. In all the cases a reduction in mechanical properties for all samples compared to PET/ITO substrate (elongation at break (240.8 ± 9.2) MPa with relative elongation at (77 ± 4)%) was observed due to the increased internal area of 20–30 µm in thickness. It should be noted that the registered maximum forces were higher for SC3 and SC13 compared to that of the neat substrate—(235.4 ± 8.1) N. It turned out that the samples covered with a single component layer showed a lower resistance to the stress at break by approximately 10 MPa than the bicomponent layer giving a sequence with a decreasing order: SC3:PC_71_BM > SC13:PC_71_BM > SC3 > SC13 > PC_71_BM. It could be explained by the structure of organic layer. PC_71_BM, due to the ellipsoidal shape of molecules does not form strong interactions between molecules on the surface of the support. The situation is different for imine, that forms flat-like surface due to the presence of flat aromatic molecules. The best properties for the imines were observed for SC3 which can form hydrogen bonding due to benzothiazole flat rings. The situation seems to be similar for SC13 with the difference that the presence of thiazole linked by single bonds to aniline and fluorobenzene gives possibility of rotation within the lateral group. The combination of imine and PC_71_BM allows for the formation of extended structures where van der Waals interactions arrange molecules in a layer with the highest strength at stress.

When analysing the elongation of the whole set of samples a different trend is seen: neat imine samples showed elongation stronger than the imine:PC_71_BM mixture and PC_71_BM gave the worst result. The use of neat imines allow for the formation of a more compact structure in which the molecules could move in the elongation direction and not completely lose the intermolecular interaction. In the case of the imine:PC_71_BM samples, the layer contains spacers (PC_71_BM) that weaken the interaction between imine molecules.

### Dielectric properties of PC_71_BM, imines and their mixture with PC_71_BM

**Figure 12:**
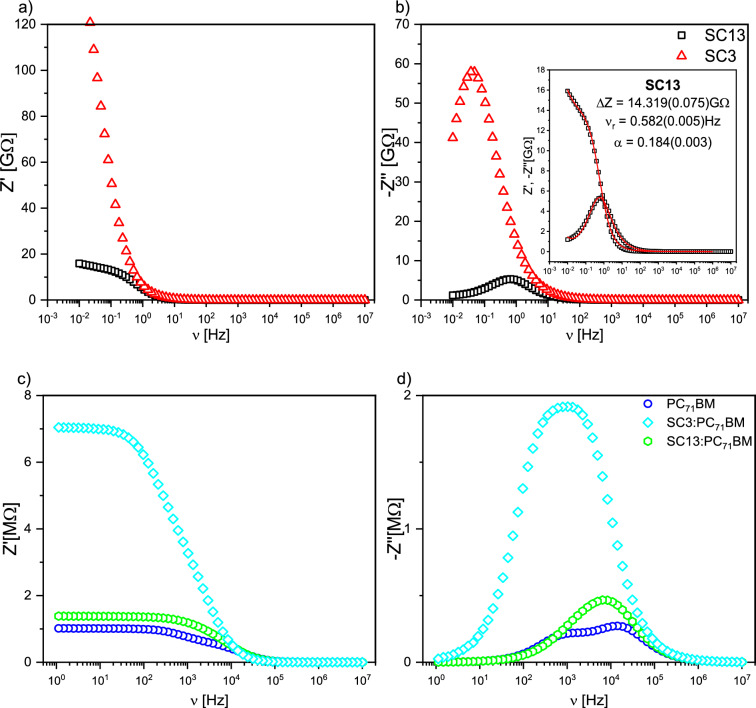
Impedance spectra for all studied systems: pristine imines SC3 and SC13 (**a**), (**b**) and PC_71_BM, SC3:PC_71_BM and SC13:PC_71_BM (**c**), (**d**). Solid lines and parameters in the inset in (**b**) are the result of fitting the Cole–Cole model to the experimental data for SC3.

As it was mentioned in the experimental part, due to fluctuations in the low frequency of εʹ(f) and εʺ(f) for the PC_71_BM sample, the measuring frequency range was limited from 1 Hz to 10 MHz for all the samples based on PC_71_BM. In our opinion these fluctuations (not observed for pristine imines) may indicate a change in conductivity character of PC_71_BM-based samples. Possibly, both the hopping mechanism and mobile ions or impurities of the fullerene derivative are responsible for the mechanism of electric conductivity and fluctuations in the mentioned frequency range. Figure 12 shows as an example the impedance spectra recorded at 172 °C for all studied systems: neat imines SC3 and SC13, PC_71_BM as well as imine:PC_71_BM mixture. The resistance (Zʹ) above 10 kHz for SC13 and SC3 is the same, while the reactance (Zʺ) for the imine SC3 is higher in the entire studied frequency range. After adding the imine SC3 to PC_71_BM, a stronger increase in Zʹ and Zʺ is seen than after adding SC13 (see Fig. 12c,d). Two phenomenological models were used: Cole–Cole and Havriliak–Negami, which can be described by the general formula:

3 $${\varepsilon }^{*}(\upomega )={\varepsilon }{\prime}(\upomega )-i\varepsilon {\prime}{\prime}(\upomega )={\varepsilon }_{\infty }+\frac{{\varepsilon }_{0}-{\varepsilon }_{\infty }}{{\left(1+{\left(i\upomega \tau \right)}^{1-\alpha }\right)}^{\beta }},$$

where ε^*^ is the complex permittivity, *ε’*(*ω*) and *ε’’*(*ω*) are real and imaginary parts of the complex permittivity, *ω* = 2πν is the angular frequency, ν—the frequency of measuring AC electric field, *ε*_0_—the low frequency limit of the permittivity, *ε*_*∞*_—the high frequency limit of the permittivity, ${\tau }_{r}=1/(2\pi {\nu }_{r})$ is the relaxation time, ${\nu }_{r}$—relaxation frequency. For symmetrical distribution of the relaxation time one can obtain the Cole–Cole model with parameters 0 < *α* < 1 and *β* = 1^44^ while for the asymmetrical distributions Havriliak–Negami model with 0 < *α* < 1 and 0 < *β* < 1^45^. The Cole–Cole model was used for the analysis of experimental data of pristine imines SC3 and SC13 while the Havriliak-Negami for systems based on the fullerene derivative PC_71_BM (pristine PC_71_BM, SC3:PC_71_BM and SC13:PC_71_BM). Inset in Fig. 12b shows the result of fitting the Cole–Cole model to the experimental data together with the fitting parameters obtained.

**Figure 13:**
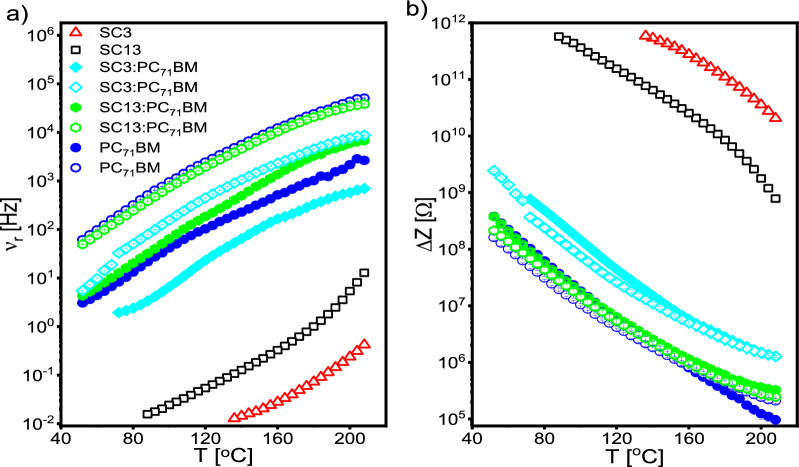
Temperature dependence of relaxation frequency (**a**) and impedance increment (**b**) of relaxation processes registered for all studied systems. Open and closed symbols denote high- and low-frequency relaxation processes, respectively.

For both imines SC3 and SC13 one relaxation process is visible in a wide temperature range (Fig. 12a,b), the relaxation frequency of which decreases with decreasing temperature and moves beyond the measurement frequency range at temperature ca. 88 and 136 °C for SC13 and SC3 imines, respectively (see Fig. 13a). The relaxation frequency of this process is always higher for imine SC13 than for imine SC3 (even about two orders of magnitude at 220 °C and one order of magnitude at about 136 °C). Considering the structure of both imines (common core and symmetry, see Fig. 1), we believe that in both imines this process has the same origin. The low relaxation frequency of this process (20–0.01 Hz) excludes its dipole character, but it may be due to the relaxation occurring at grain boundaries (the studied imines in the form of pellets consisted of different sizes of compacted crystallites). In turn, taking into account the temperature dependence of the relaxation frequency, it cannot be ruled out that this process involves certain relaxations at the electrode-sample interface. Resolving this issue requires further research.

On the other hand, for investigated systems based on PC_71_BM, two closely located relaxation processes are visible in the range of higher frequencies than those for pure imines (Fig. 12c,d). The relaxation frequencies of these processes decrease rapidly with decreasing temperature (Fig. 13a). Interestingly, after the addition of imines to the PC_71_BM, the process registered for pure imines is not observed. The probably low ratio of imine to PC_71_BM in the system imine:PC_71_BM (1:8) and good distribution of imine in the PC_71_BM prevents the imine grains from touching each other, therefore the relaxation at the borders of neighboring grains does not take place. This confirms our earlier supposition of the origin of this process in the imines.

Interestingly, the addition of imine to the PC_71_BM strongly modified the relaxation frequencies of both processes registered in pure PC_71_BM. In the case of the low-frequency process, the addition of the imine SC13 caused a shift to higher frequencies while the addition of the SC3 imine to lower frequencies, with the effect of the imine SC3 on this process being more significant. On the other hand, the addition of imine decreases the relaxation frequencies of the high-frequency process, and the effect of imine SC3 is also more significant.

The impedance increment ∆Z for the imine SC13 is smaller than for SC3 in the entire temperature range (Fig. 13b). In turn, ∆Z for both relaxation processes in PC_71_BM-based systems are comparable, but they change after the addition of imine (Fig. 13b). The addition of the imine SC13 causes a significant increase of ∆Z of the low-frequency process above the temperature of 150 °C, and at the same time an increase of ∆Z of the high-frequency process below this temperature. On the other hand, ∆Z of both relaxation processes increases after addition of imine SC3 (Fig. 13b).

As mentioned earlier, the dominant contribution to the molecule's PC_71_BM dipole moment comes from the fullerene core^46^. Thus, under the influence of an external electric field, a moment of force acts on the molecule, which orders the PC_71_BM molecule in accordance with the external electric field, and the high-frequency relaxation process in PC_71_BM-based systems has a dipole character.

Dhibi et al*.* for thin films of PCDTBT:PC_71_BM (1:4) recorded two relaxation processes (at 10^2^ and 10^6^ Hz) in dielectric spectra, with the high-frequency process interpreted as a dipole reorientation^47^. This confirms our supposition that the high-frequency process observed in the PC_71_BM-based systems is associated with dipole reorientation, and the differences in relaxation frequencies may result from the shift of dielectric and impedance spectra (here impedance spectra), sample thickness (approx. 100 nm and approx. 300 μm) and illuminating PCDTBT:PC_71_BM samples during measurements.

**Figure 14:**
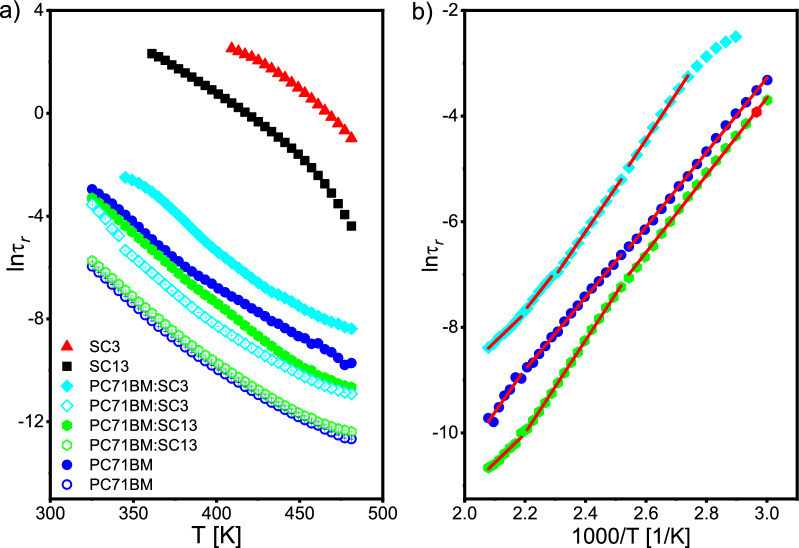
Temperature dependence of the relaxation time for all processes registered in the studied system (**a**) and Arrhenius plot for low-frequency process registered in systems based on PC_71_BM. Solid lines in (**b**) are results of fitting Arrhenius equation to the temperature dependence of relaxation time (Eq. 4).

Due to the non-Arrhenius behavior of the relaxation processes revealed in the pristine imines SC3 and SC13 (Fig. 13), the activation energy ∆*E* was determined only for systems based on PC_71_BM according to the Arrhenius Eq. (4):

4 $${\tau }_{r}\left(T\right)={\tau }_{0}\mathrm{exp}\left(\frac{\Delta E}{RT}\right)$$

where ${\tau }_{0}$ is constant, $\Delta E$—the activation energy and $R$—the gas constant. Only for the SC3:PC_71_BM system for the high-frequency process, a change in the slope was observed on the Arrhenius plot at a temperature of about 70 °C, and therefore two activation energies for this process were determined (in the temperature range of 68–52 °C and 208–72 °C) in this system (Fig. 14). The obtained activation energies are summarized in Table 4. It can be seen that the addition of imine SC13 does not affect the activation energy of the high-frequency relaxation process (similarly, the relaxation frequency is slightly modified). On the other hand, after adding imine SC3, the activation energy increases (relaxation frequency is about an order of magnitude lower than for neat PC_71_BM in the entire temperature range).

**Table 4:** Activation energy of high- and low-frequency relaxation processes revealed in systems based on PC_71_BM.

Sample	High-frequency process	Low-frequency process
	∆E[kJ/mol]	∆E[kJ/mol]
PC_71_BM	57.35 ± 0.17 (208–52 °C)	68.43 ± 8.20 (208–184 °C)
		56.94 ± 0.35 (180–124 °C)
		58.55 ± 0.55 (120–52 °C)
SC13:PC_71_BM	56.96 ± 0.31 (208–52 °C)	45.85 ± 3.30 (208–184 °C)
		73.00 ± 0.36 (180–124 °C)
		60.83 ± 0.69 (120–52 °C)
SC3:PC_71_BM	58.70 ± 0.52 (208–72 °C)	45.57 ± 2.08 (208–184 °C)
		69.62 ± 0.21 (180–164 °C)
	70.94 ± 2.60 (68–52 °C)	60.62 ± 1.89 (160–124 °C)
		72.53 ± 1.24 (120–52 °C)

On the other hand, for the low frequency process, three linear regions of ln(τ(1/*T*)) are visible on the Arrhenius plot, and therefore three values of activation energy ∆*E* were determined. In the high temperature range (208–184 °C), the addition of both imines reduces the activation energy in the same way (from ca. 68 to 45 kJ/mol), and in the remaining temperature range the activation energy increases after the addition of imines, with SC3 imine having a greater effect (see Table 4).

Based on the activation energies of the low- and high-frequency process, it may be assumed that there are two different populations of molecules in the PC_71_BM and these two relaxation processes from the molecular point of view are identical, but the dipole reorientation occurs faster for one population of molecules and slower for the other. This behaviour could suggest that PC_71_BM molecules with a permanent dipole moment are oriented antiparallel. However, this hypothesis requires further research, e.g. using secondary ion mass spectroscopy.

On the other hand, the strong effect of SC3 imine and no effect of SC13 imine on both relaxation time and activation energy ∆*E* of the high-frequency process may prove that the SC3 molecule is much more rigid. Furthermore, different mobility and different orientation of SC13 and SC3 molecules relative to the PC_71_BM cannot be excluded.

The mass of imines in the SC13:PC_71_BM and SC3:PC_71_BM samples is the same (2.78 mg). However, they have different molar masses (SC13: 809.02 g/mol, SC3: 568.78 g/mol) and the amount of imine molecules N introduced into the PC_71_BM is much bigger for SC3 than SC13:

5 $$N=\frac{m{N}_{A}}{M}$$

where ${N}_{A}$—Avogadro constant, $M$—molar mass, $m$—mass of the sample. Since $N$ is 2.069∙10^18^ and 2.943∙10^18^ molecules for SC13 and SC3, respectively, the number of imine molecules introduced has probably a strong influence on the observed relaxation processes in the PC_71_BM, especially on the high-frequency process. It is possible that adsorption of imine molecules to PC_71_BM takes place, and the coefficient of kinetic friction between PC_71_BM and imine will be different for SC13 and SC3 imines. The change of the activation energy after the addition of the imines indicates that the friction coefficient between the PC_71_BM and SC13 molecules is lower than that of PC_71_BM and SC3. Thus, relaxation in the SC13:PC71BM system will be characterized by a smaller energy barrier (lower ∆E). The kinetic friction coefficient may change with decreasing temperature for both imines, which explains the changes of the activation energy of a given process. Understanding the interaction at the molecular level between the PC_71_BM and admixtures (SC13 and SC3) requires further research.

Joshi reports that grain boundary relaxation processes are very sensitive to doping^48^, e.g. for undoped crystal of lead tartrate two relaxation processes are visible: the grain boundary process (boundary effect) and the grain process (grain effect), while the grain boundary process disappears after doping. Both relaxation processes (grain effect and grain boundary effect) were also observed for the polycrystalline inorganic semiconductor Pr_2_ZnMNO_2_^49^. In addition, the relaxation frequency associated with grain boundaries can take different values depending on the material. At the grain boundaries, the material is discontinuous, which causes polarization at such an interface. It is possible that in the samples of imines there are small spaces filled with air on the micrometer scale. In this case, in addition to the grain-to-grain interface, there is a grain-to-air interface. Such interfaces were considered by Steeman and van Turnhout in poly(vinyl chloride) samples^50^. It turns out that such parasitic air spaces provide an additional contribution to the relaxation process associated with grain-grain interphase. The presence of small air spaces in the studied SC13 and SC3 samples is possible due to the irregular shapes of the crystals used to form pastilles.

**Figure 15:**
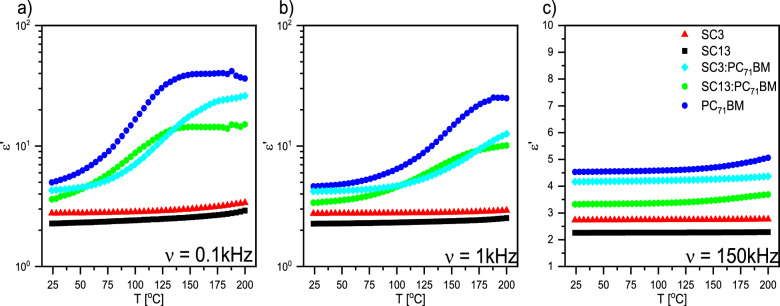
Temperature dependence of the dielectric dispersion at chosen frequencies: (**a**) 0.1 kHz, (**b**) 1 kHz and (**c**) 150 kHz.

Figure 15 presents dielectric dispersion as a function of temperature εʹ(T) for selected frequencies. The dielectric dispersion determined by us at the high frequency limit for PC_71_BM coincides with that determined by Asandulesa et al*.*^51^. It is known that relative permittivity is a measure of the polarizability of a dielectric, therefore a material with high permittivity stores more electric energy. As is seen, imines polarize much weaker than PC_71_BM at selected frequencies in the entire temperature range, and εʹ for SC13 and SC3 practically does not depend on temperature and frequency, and is greater for SC3 than SC13. The PC_71_BM, on the other hand, polarizes more at higher temperatures and low frequencies (0.1 and 1 kHz), while for higher frequency (150 kHz) εʹ is almost constant. After adding imines to PC_71_BM, εʹ decreases over the entire temperature range, with εʹ(T) behaving irregularly for the frequencies 0.1 and 1 kHz (being higher for SC3:PC_71_BM in one temperature range and higher for SC13:PC_71_BM in another). Although this proves the complex behavior at the molecular level, it gives the possibility of designing systems for storing electricity depending on the operating temperature range, which is interesting from the point of view of applications.

**Figure 16:**
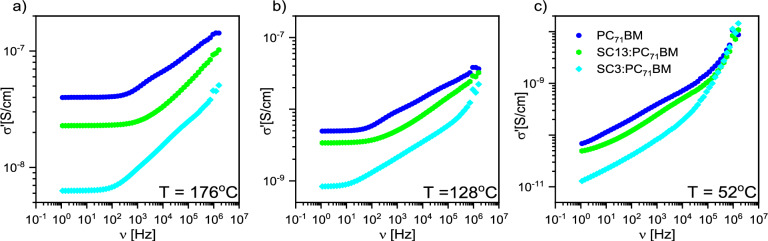
Frequency dependence of the conductivity at chosen temperature: (**a**) 176 °C, (**b**) 128 °C, (**c**) 52 °C.

From the application point of view, an important aspect is to know the effect of SC13 and SC3 imines on the conductivity of the PC_71_BM. Figure 16 presents the frequency dependence of the real part σ* for selected temperatures. It can be seen that σʹ decreases for all systems in the low-temperature region. The addition of both imines SC3 and SC13 lowers the value of σ' in the entire frequency range for all temperatures, with the effect of imine SC3 being much stronger. In the high-temperature region, the common feature of all three systems is the constant value of σ' (plateau) in the low-frequency region (up to about 100 Hz). This plateau becomes broader with increasing temperature, which was also observed by Asandulesa et al*.* for polymer PTB7 and PTB7:PC_71_BM system^51^.

It is known that there are two conduction mechanisms in semiconductors: band conduction (BC) and hopping (nearest-neighbor hopping—NNH and variable-range hopping—VRH)^52,53^. All types of conduction can occur simultaneously, but the type that is associated with the least resistance to the carriers predominates. It was also shown that the doping of semiconductors can change the dominant type of conduction, e.g. by increasing the concentration of the Al dopant in the 4H-SiC layer, Matsuura et al*.* systematically changed the BC and NNH dominating at high and low temperatures to VRH, which became dominant over the entire temperature range for the highest concentrations of Al^52^.

Since PC_71_BM is an organic semiconductor material (E_HOMO_ = − 5.9 eV, E_LUMO_ = − 3.9 eV), the electric conductivity can be described by the hopping conduction theory between localized states. According to this theory in a partially disordered material, the probability of hopping on a charge carrier $W$ between two sites $R$ distant is given by Eq. (6):

6 $$W\propto exp\left(-2\alpha R\right)exp\left(-\frac{{E}_{A}}{{k}_{B}T}\right)$$

where 1/$\alpha$—attenuation length, ${E}_{A}$—energy barrier between the energy states of two places^54^. At low temperatures, hopping is possible not only between the nearest neighbors but also between distant places where the hopping frequency is low. To describe the transport of carriers in disordered semiconductors and amorphous solids, the variable-range hopping VRH model is used, in which the electric conductivity σ can be described as Eq. (7):

7 $$\sigma \left(T\right)={\sigma }_{VRH}\mathrm{exp}\left(-{\left(\frac{{T}_{0}}{T}\right)}^{\frac{1}{\beta }}\right)$$

where ${\sigma }_{VRH}$ is pre-exponential factor, depending on the model (${\sigma }_{MVRH}$ or ${\sigma }_{ESVRH}$ in the case of the Mott or the Efros-Shklovskii model), $T$—temperature, ${T}_{0}$—fitting parameter, and $\beta$—parameter depending on the model used ($\beta =4$ in Mott model and $\beta =2$ in Efros-Shklovskii model). The Mott model describes low-temperature electric conductivity in highly disordered systems with localized charge carrier states. In turn, band conduction is the dominant mechanism at high temperatures. In addition, at lower temperatures, thermal activation makes hopping between nearest neighbors very likely. In both cases, the specific electric conductivity satisfies the standard Arrhenius relations (8–9):

8 $$\sigma \left(T\right)={\sigma }_{BC}\mathrm{exp}\left(-\frac{{E}_{A,BC}}{RT}\right)$$

9 $$\sigma \left(T\right)={\sigma }_{NNH}\mathrm{exp}\left(-\frac{{E}_{A,NNH}}{RT}\right)$$

where ${E}_{A,BC}$ and ${E}_{A,NNH}$ are the activation energies when the band conduction or NNH conduction mechanism is dominant.

**Figure 17:**
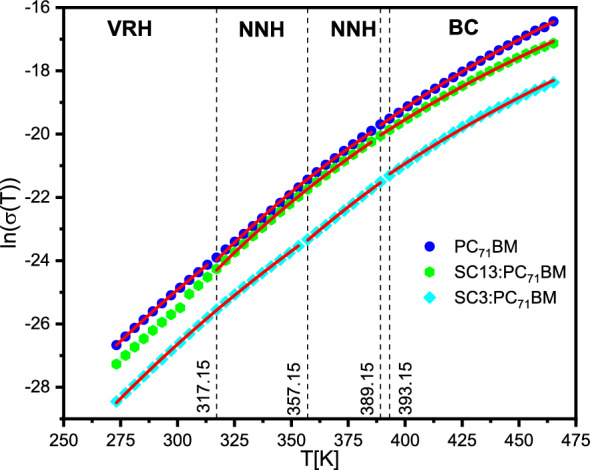
Arrhenius plot for systems based on PC_71_BM. Solid lines are results of fitting various models to the experimental data. The vertical dashed lines indicate the temperature of model used to describe the conductivity.

Figure 17 presents the Arrhenius plots for the quasi-static conductivity σ_DC_ (at ν = 1.123 Hz) for PC_71_BM-based systems. It is seen that the σ_DC_ decreases after doping the PC_71_BM with SC13 or SC3 imines in the entire temperature range. Although the conductivity of PC_71_BM decreases after doping with imines SC13 and SC3 (contrary to the case of CoSb_3_ doped with Ni atoms^53^), the increase in σ' with increasing temperature proves the semiconducting nature of all studied systems based on PC_71_BM. There are basically three regions with different conduction mechanisms for each sample: high temperature with band conduction (BC), intermediate temperature with NNH hopping and low temperature with VRH. Based on the fitted conduction models, the activation energies for the BC and NNH mechanism and the values of the inverse of the $\beta$ exponent for VRH model were determined (Table 5).

**Table 5:** Activation energy and inverse of parameter *β* obtained by fitting Eqs. 7,9.

Sample	BC	NNH	VRH
	*E*_A,BC_ [kJ/mol]	*E*_A,NNH_ [kJ/mol]	1/*β*
PC_71_BM	64.82 ± 0.17	60.33 ± 0.45	0.232 ± 0.066
SC13:PC_71_BM	58.74 ± 0.36	60.40 ± 0.20	–
SC3:PC_71_BM	62.42 ± 0.58	65.64 ± 0.63	0.260 ± 0.079

As it is seen from Table 5, both imines SC13 and SC3 decrease the activation energy of the BC conduction mechanism, while the imine SC13 does not change and the imine SC3 increases the activation energy of the NNH conduction mechanism. For the PC_71_BM ${E}_{A,NNH}$ < ${E}_{A,BC}$, and for the doped mixture, the opposite is true, which proves that two conduction mechanisms NNH and BC or NNH and VRH are dominant here. The change of the conduction mechanism along with the change of temperature are clearly visible for the PC_71_BM and SC3:PC_71_BM systems (distinct BC, NNH and VRH temperature regions). In turn, the parameter 1/*β* (obtained by fitting the VRH model) is within the uncertainty limit equal to 0.25 for both PC_71_BM and SC3:PC_71_BM, which proves that the Mott mechanism (MVRH) occurs in the VRH region. In turn, for the SC13:PC_71_BM system, it was not possible to fit the VRH model, which suggests that the Efros-Shklovskii (ESVRH) hopping mechanism occurs in this area. However, it cannot be ruled out that in this area there is no dominant single hopping mechanism, but it is mixed of the MVRH:ESVRH type. These hypotheses require further research at lower temperatures.

## Conclusions

The dielectric, impedance and electric conductivity as well as optical, thermal and mechanical studies were performed for pristine imines (SC13, SC3), PC_71_BM and for two-component systems SC3:PC_71_BM and SC13:PC_71_BM. We found that the relaxation processes registered for both imines can be attributed to grain-boundary relaxation, and that the imine SC13 has much lower resistance than SC3. For systems based on PC_71_BM, a greater effect of imine SC3 than SC13 on the properties of PC_71_BM was noted: the resistance increases about 7 times after the addition of imine SC3 and only by the factor of 1.5 after adding of SC13. Two relaxation processes have been distinguished for the systems of doped PC_71_BM, whereas no processes characteristic for imines were found. The addition of imine SC13 practically does not affect the high-frequency relaxation process in PC_71_BM, but it accelerates the low-frequency one. In turn, the addition of imine SC3 significantly slows down both relaxation processes in PC_71_BM, by increasing their activation barrier. The high-frequency process has a dipole character, while the low-frequency one can be associated with the grain boundary relaxation or two different molecule population of PC_71_BM for both systems SC3:PC_71_BM and SC13:PC_71_BM. As expected, the addition of dielectric SC3 and SC13 decrease the electric conductivity of the doped superconductor. On cooling, the value of σ'(ν) decreases for PC_71_BM and for the systems based on it, which proves that the semiconductor character after adding SC13 and SC3 is preserved (in the ratio 1:8 imine:PC_71_BM). The quasi-DC conduction mechanisms in the PC_71_BM and systems based on it were also determined. In the high-temperature range, the BC conduction mechanism is dominant, in the intermediate temperature for PC_71_BM, the NNH mechanism is dominant, while in SC3 and SC13:PC_71_BM two mechanisms, NNH and BC or NNH and VRH, are likely. In the low- temperature range for PC_71_BM and SC3:PC_71_BM, the conduction is governed by the Mott hopping mechanism (MVRH), while for SC13:PC_71_BM the mechanism could not be clearly determined.

The thermoelectric studies revealed a thermal degradation of the PET-supported samples above 8 V, and the worst performance of imine SC3. It was also observed that the addition of PC_71_BM to the active layer with imine improves its conductive properties. Moreover, the samples continue to conduct in high-temperature range reaching around 453 K (180 °C), close to the critical point for the substrate starting to significantly deform at this temperature.

The mechanical studies revealed better properties of the samples containing imines compared to neat PC_71_BM. It was noticed that the addition of fullerene derivative to imine resulted in an improvement in breaking strength reaching in the best case more than 200 MPa for SC3:PC_71_BM. The presence of fullerene had an adverse effect decreasing the elongation of substrate. The strong strain generated by the thermal expansion of the substrate, and by its deformations for the flexible photovoltaic panels, prompted the high-pressure studies. They revealed bathochromic shifts for all PC_71_BM, SC3 and SC13 compounds. The pressure dependence of PC_71_BM and SC13 is linear, while an anomalous absorbance of SC3 may be associated with a solid–solid phase transition in this compound. The rates of pressure dependence calculated for the 0–3 GPa range are 21 nm/GPa for PC_71_BM, 28 nm/GPa for SC3 and 22 nm/GPa for SC13. These band-gap *E*_g_ reduction, reflecting the compressed intermolecular interactions, is advantageous for the performance of the photovoltaic devices, but it can be also applied for monitoring the structural stability of these compounds on one hand and for monitoring the mechanical strain and other conditions of the devices on the other.

## Data Availability

The datasets used and/or analysed during the current study available from the corresponding author on reasonable request.
