# Author Correction: A new look at imines and their mixture with PC_71_BM for organic, flexible photovoltaics

**DOI:** 10.1038/s41598-023-42036-x

**Published:** 2023-09-13

**Authors:** Krzysztof A. Bogdanowicz, Sebastian Lalik, Paulina Ratajczyk, Andrzej Katrusiak, Piotr Krysiak, Agnieszka I. Pawłowska, Monika Marzec, Agnieszka Iwan

**Affiliations:** 1 Military Institute of Engineer Technology, Obornicka 136, 50-961 Wrocław, Poland; 2 Institute of Physics, Jagiellonian University, Łojasiewicza 11, 30-348 Kraków, Poland; 3 Faculty of Chemistry, Adam Mickiewicz University, Uniwersytetu Poznańskiego 8, 61-614 Poznań, Poland

Correction to: *Scientific Reports* 10.1038/s41598-023-38978-x, published online 14 August 2023

The original version of this Article contained an error in the spelling of the author Paulina Ratajczyk, which was incorrectly given as Paulina Ratajczak.

The original Article has been corrected.

